# Repetitive Transcranial Magnetic Stimulation on the Affected Hemisphere Enhances Hand Functional Recovery in Subacute Adult Stroke Patients: A Randomized Trial

**DOI:** 10.3389/fnagi.2021.636184

**Published:** 2021-05-19

**Authors:** Yawen Yang, Huijuan Pan, Wenxiu Pan, Yang Liu, Xiaohui Song, Chuanxin M. Niu, Wuwei Feng, Jixian Wang, Qing Xie

**Affiliations:** ^1^Department of Rehabilitation Medicine, Ruijin Hospital, Shanghai Jiao Tong University School of Medicine, Shanghai, China; ^2^Department of Rehabilitation Medicine, Shanghai Ruijin Rehabilitation Hospital, Shanghai, China; ^3^Department of Neurology, Duke University School of Medicine, Durham, NC, United States

**Keywords:** hand function, neuro-modulation, stroke, transcranial magnetic stimulation, rehabilitation

## Abstract

**Objectives:** Either motor training or repetitive transcranial magnetic stimulation (rTMS) could modulate the neural plasticity after stroke. Therefore, synchronizing the two interventions may optimize the efficiency of recovery. In the present study, we aim to investigate the effect of rTMS along with hand grip training on the neurobehavioral and hand functional recovery in one cohort of subacute stroke patients.

**Methods:** Thirty-nine stroke patients were enrolled in a single-center, single-blinded, randomized clinical trial. We tested different intervention effects of rTMS and hand grip training (group A), rTMS alone (group B), and hand grip training alone (group C). For the rTMS-treated groups, patients received 10 consecutive sessions of 5-Hz stimulation over the affected hemisphere with 750 pulses. Jebsen–Taylor Hand Function Test (JTHFT), Fugl-Meyer assessment of upper extremity (FMA-UE), grip strength, modified Barthel index (mBI), and ipsilesional motor evoked potential (iMEP) latency were assessed and compared across the groups.

**Results:** We found that only rTMS along with hand grip training group all improved in JTHFT, FMA-UE, grip strength, and mBI (*p* ≤ 0.01) compared with the baseline among the three groups. Furthermore, this study demonstrated that rTMS plus hand grip training had much better results in improvement of neurobehavioral outcomes compared to the rTMS alone- and hand grip training alone-treated patients (*p* < 0.05). However, no significant differences were detected in neurophysiologic outcome between intra-groups and inter-groups (*p* > 0.05).

**Conclusion:** These proof-of-concept results suggested that rTMS alone with hand grip training was a unique approach to promote hand functional recovery in stroke patients. It provided important information to design a large-scale multi-center clinical trial to further demonstrate the efficiency of the combination of central and peripheral stimulation.

**Clinical Trial Registration:** http://www.chictr.org.cn (#ChiCTR1900023443).

## Introduction

Despite intensive neurorehabilitation efforts, hand- and finger-related functional abilities remain unsatisfactory following a neurological event. Studies demonstrated that 27% of stroke patients lose integral hand function due to hemiplegia (Fischer et al., 2007). It was no surprise that one of the most commonly expressed goals of individuals who sustained stroke was to engage in neurorehabilitation interventions that could enhance hand function. For stroke survivors, therefore, improving related hand functional abilities and promoting the hand function become crucial for optimal social participation and daily life (Higgins et al., 2013). Previous studies demonstrated that the establishment of neural plasticity after stroke is essential for the motor recovery (Hermann and Chopp, 2012). Furthermore, motor training is capable of promoting neuroplasticity to attenuate the motor dysfunction in stroke patients (Kleim et al., 2003; Adkins et al., 2006). For instance, hand grip training could be beneficial for the finger flexor and extensor tendon to recruit more motor units and improve the innervation and functional neuroplasticity, which played an important role in the motor recovery of stroke survivors (Murphy and Corbett, 2009). From the view of previous trials, hand functional training could regulate the neuronal excitatory input in the nerve reflex circuits and accelerate the process of reorganization of brain connectivity and neuroplasticity (Wolf et al., 2006; Buma et al., 2010).

Repetitive transcranial magnetic stimulation (rTMS) could re-balance inter-hemisphere inhibition (IHI) by either up-regulating or down-regulating cortical excitability (Nowak et al., 2009). The influence of stroke is mainly restricted to the affected hemisphere, and facilitating affected M1 directly might produce more enhancement of motor recovery than suppressing the unaffected M1 excitability (McDonnell and Stinear, 2017). Despite rTMS representing an ideal approach to promote neural plasticity (Fregni et al., 2006; Bolognini et al., 2009), whether the combination of rTMS and motor training could enhance the therapeutic effect and prolong the effective period was largely unknown. The application of motor training combined with rTMS over targeted motor cortex in healthy subjects and chronic stroke patients showed strong support both in the concept (Morris, 1999) and initial experimental evidence (Bütefisch et al., 2004; Izumi et al., 2008; Massie et al., 2013a). However, the brain tissue repair process was more complicated especially in the subacute phase. Providing corresponding proof was necessary and timely.

We aim to investigate the combined effect of high-frequency rTMS (HF-rTMS) and hand grip training on the impaired hand functional recovery in stroke survivors. We hypothesized that HF-rTMS, along with hand grip training, induces significant hand functional recovery in subacute stroke patients as compared to the controls, which was related to the establishment of functional neuroplasticity.

## Materials and Methods

### General Information

The study protocol was approved by the Institutional Ethics Review Board, Shanghai Ruijin Rehabilitation Hospital, Shanghai, China. The clinical trial was registered in the Chinese Clinical Trial Registry (ChiCTR) with the registration number ChiCTR1900023443. All enrolled patients signed the informed consent prior to the enrollment to this study.

Thirty-nine hemiparetic stroke patients were enrolled at Shanghai Ruijin Rehabilitation Hospital from March 2019 to December 2019. The criteria of enrollment were as follows: (1) confirmed clinical stroke diagnosis, which was based on the Fourth National Conference on Cerebrovascular Diseases in 1995; (2) the first ischemic or hemorrhagic stroke confirmed by CT or MRI scans; (3) 1 to 6 months from stroke onset; (4) age from 40 to 75 years old; (5) Brunnstrom of hemiplegic upper limb and hand staging from 4 to 5; (6) Mini Mental State Examination (MMSE) > 20/30; and (7) informed consent to the study and signed the consent of rTMS. The exclusion criteria were as follows: (1) uncontrolled hypertension; (2) history of seizure or using epileptic drugs either before or after stroke; (3) heart, lung, liver, kidney, or other essential organ functional decline or failure; (4) aphasia, ipsilateral neglect, hemianopia, or affective disorder that affects participant’s ability to comply with study procedure; and (5) known risk factors for TMS such as having a pacemaker, intracranially implanted metal, skull defects, etc.

This study was a randomized, well-designed and controlled, prospective clinical trial conducted at a single center, which included four phases: (1) baseline evaluation, (2) randomization, (3) intervention, and (4) post-intervention evaluation. Thirty-nine stroke patients were randomly assigned by a number generated from a computer randomization table. All of the participants who met the criteria were assigned to three groups (groups A, B, and C) on the basis of the random numbers. Thirty-nine stroke patients with mild motor dysfunction recruited from inpatients and outpatients were randomized into rTMS with hand grip training (group A), rTMS alone (group B), and hand grip training alone (group C). The clinical demographic characteristics of participants are listed in Table 1.

**TABLE 1:** Demographic and clinical characteristics.

	Group A	Group B	Group C	*p*
	*n* = 12	*n* = 14	*n* = 13	
Gender—m/f	10/2	10/4	8/5	0.481^a^
Age (years)	64 ± 8	61 ± 10	64 ± 8	0.724^b^
Stroke Onset (days)	64 ± 23	79 ± 43	75 ± 49	0.820^b^
Stroke—i/h	10/2	11/3	10/3	0.919^a^
Lesion location				0.176^a^
Cortex	0	0	2	
Subcortex	9	13	10	
Both	3	1	1	
FMA-UE	47 ± 6	47 ± 8	47 ± 7	0.990^c^

*Group A, high-frequency rTMS during hand grip training; Group B, high-frequency rTMS alone; Group C, hand grip training alone; rTMS, repetitive transcranial magnetic stimulation. m, male; f, female; i, ischemic; h, hemorrhagic; FMA-UE, Fugl–Meyer assessment of upper extremity; rTMS, repetitive transcranial magnetic stimulation. Data are mean ± SD. *p*^*a*^, chi-squared test; *p*^*b*^, nonparametric Kruskal–Wallis *H* test; *p*,: one-way ANOVA.*

### Intervention

Intervention lasted 10 days with two interventions: (1) 5 min of real/sham 5-Hz rTMS. Each session consisted of 5-Hz rTMS for 1 s, which was both preceded and followed by a resting period of 1 s (total time = 150 s); and (2) 5 min of hand grip training. The hand grip training is composed of a repeated 1-Hz rhythmic voluntary grip by holding a ball. Apart from these, all of the participants received the conventional rehabilitation, which involved physical therapy and occupational therapy for 120 min daily for 10 sessions (5 days/week for 2 weeks). The interventions were required to be steadily implemented after conventional rehabilitation. It was worth mentioning that the practice of standardized conventional rehabilitation was requested to avoid involving evaluation projects.

During each intervention, patients seated in a comfortable and adjustable chair with headrest and armrests and took a comfortable supine position, maintaining the head and neck without displacement. Patients were instructed not to move their heads during the treatment period. The upper limb of the unaffected side was naturally placed on the armrest of the seat, and the upper limb of the affected side was placed on the side of the body.

A TMS device code with CCY-IV (Yi Ruide Company, Wuhan, China) with a 90-mm figure-of-eight coil was utilized for rTMS. rTMS intervention and evaluation were conducted in a quiet room and implemented by a trained research staff member. Motor evoked potential (MEP) signals were recorded by an electromyography (EMG) instrument that was connected to the stimulator. Ag-AgCl surface electrodes were firstly placed over the abductor pollicis brevis (APB) muscle of the unaffected hand. rTMS was applied over M1 of the unaffected hemisphere according to electroencephalogram (EEG) 10/20, where the MEP was elicited. The resting motor threshold (RMT) of APB of the unaffected upper extremity was determined by decreasing or increasing the intensity from 50% in a stepwise manner. Plus, the RMT was defined as the minimal stimulus intensity that produced a MEP response of at least 50 μV amplitude with the APB muscle at rest in at least 5 of 10 subsequent stimulations (Rossini et al., 2015). We selected the optimal stimulation site (“hot spot”) in the unaffected hemisphere where the largest MEP could be consistently evoked, with the APB muscle at rest. Following that, the electrodes were placed on APB muscle of the affected hand when detecting the MEP signal of the injured hemisphere. If no MEP could be detected when stimulating over the affected M1, the optimal stimulation site was defined as the symmetric location to the “hot spot” of the unaffected hemisphere according to EEG 10/20. The MEP latency and amplitude of the targeted muscle were measured as the period (ms) between stimulus onset and the start of the largest MEP and their peak-to-peak (mV). The parameters of stimulation were 5 Hz, 100% RMT, and 750 pulses, for a total of 10 sessions (5 days/week for 2 weeks). The sham stimulation was conducted with the coil placed rotated 90°away from the scalp resulting in no current generated in the brain.

### Evaluation

Measurements of Jebsen–Taylor Hand Function Test (JTHFT), Fugl-Meyer assessment of upper extremity (FMA-UE), grip strength, modified Barthel Index (mBI), and ipsilesional motor evoked potential (iMEP) latency were assessed at baseline and post-intervention. Primary outcome was administered by a skilled clinician to evaluate the total time of accomplishing the tasks of JTHFT. Secondary outcomes included arm and hand function, force, daily living ability, and cortical excitability that were recorded by the same evaluator.

#### Jebsen–Taylor Hand Function Test

The JTHFT assesses hand dexterity and consists of seven different subtasks: (1) writing a sentence, (2) turning cards, (3) moving small common objects, (4) simulating feeding, (5) stacking checkers, (6) picking up large light cans, and (7) moving heavy cans (Jebsen et al., 1969). The participants performed each task with the affected limbs and the duration of each task from onset (lifting hand from table) to the completion using a stopwatch to record in seconds (the maximal duration is 120 s per task) and summated as the total score (Radder et al., 2019). The result of JTHFT could explain three factors in relation to basic fine motor skills: motor coordination, speed of movement, and grip force scaling. Motor coordination includes tasks 1, 4, and 5. The speed of movement consists of tasks 2, 3, and 6. Task 7 reflects grip force scaling (Allgower and Hermsdorfer, 2017).

#### Fugl-Meyer Assessment of Upper Extremity

The FMA-UE included 33 items, and the score range was 0–66. Among them, the hand function part accounted for 24 points. Each item was recorded in an ordinal scale (0 represents severe impairment and 2 represents no impairment) (Gladstone et al., 2002).

#### Grip Strength

Grip strength was measured by a hand dynamometer (Mathiowetz et al., 1985). The subjects were requested to squeeze the handgrip of the dynamometer maximally for 5 s. Each participant had three attempts, and the interval between the attempts was at least 60 s rest. The highest grip strength was recorded for analysis in kilograms.

#### Modified Barthel Index

The mBI was recorded, which included personal hygiene, self-bathing, feeding, using the toilet, stair climbing, getting dressed, bowel control, bladder control, chair/bed transfer, and ambulation (Shah et al., 1989). The total score is 100 points.

#### iMEP Latency

The MEP latency reflected the conduction time for neural impulses from the cortex to peripheral muscles and suggested cortical excitability (Bestmann and Krakauer, 2015). The target peripheral muscle in this study was APB. The MEP latency of the targeted muscle was measured as the period (ms) between stimulus onset and the start of the largest MEP. We selected five MEPs per subject with stable waveforms and calculated the mean of the iMEP latency as the measurement of cortical excitability.

### Statistical Analysis

According to the previous HF-rTMS studies, they suggested the sample size of *n* = 8 for each group to be adequate (Gladstone et al., 2002; Guo et al., 2016). Based on our research sample, we calculated the power of test statistic and the value was 0.84 (β = 0.16). Statistical analysis was performed in SPSS version 23.0. Shapiro-Wilk test was used to evaluate whether the assessment scores were normally distributed. Chi-squared test was used for categorical variables while one-way ANOVA or the nonparametric Kruskal–Wallis *H* test was utilized for continuous variables to compare among the three groups. A paired *t* test or Wilcoxon rank-sum test was performed to compare the values between baseline and post-intervention within each group. The change of FMA-UE and grip strength were analyzed by one-way ANOVA, while JTHFT, mBI, and iMEP latency changes were compared by nonparametric Kruskal–Wallis H test to determine the between-group differences. Data were presented as means ± standard deviation. *p* < 0.05 was considered statistically significant.

## Results

**FIGURE 1:**
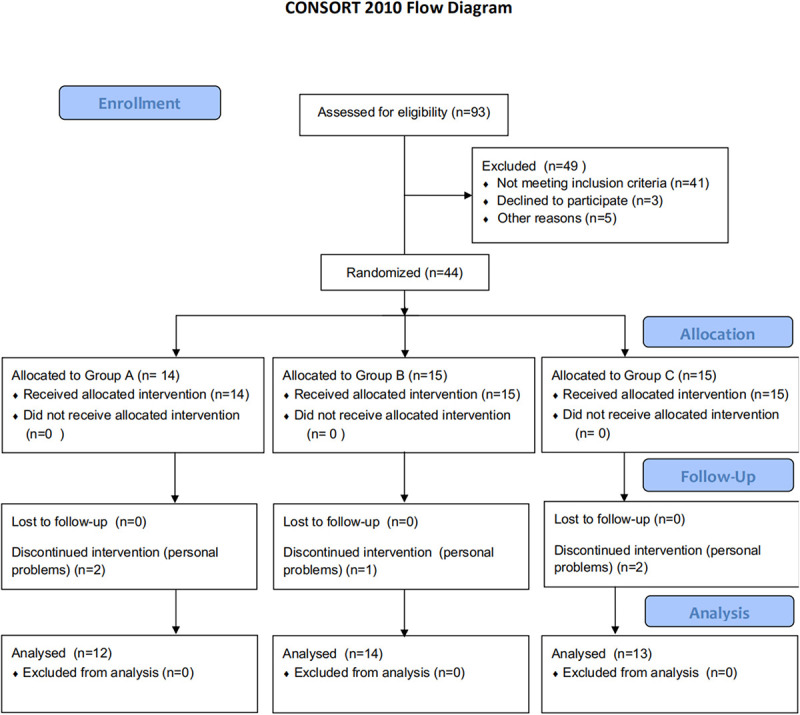
Flowchart of the trial. Group A: high-frequency rTMS during hand grip training; Group B: high-frequency rTMS alone; Group C: hand grip training alone; rTMS: repetitive transcranial magnetic stimulation. CONSORT flow diagram illustrates recruitments, group allocation, follow-up, and analysis.

Forty-four patients were assigned randomly to group A, B, or C. Five patients dropped out of the trial due to personal problems. Finally, thirty-nine patients completed the interventions without incident, severe side effects, or discomfort, indicating that the procedure was well tolerated (Figure 1). No significant differences in clinic–demographic characteristics and FMA-UE score were presented among the groups (Table 1).

### Behavioral Measures

#### Jebsen–Taylor Hand Function Test

**FIGURE 2:**
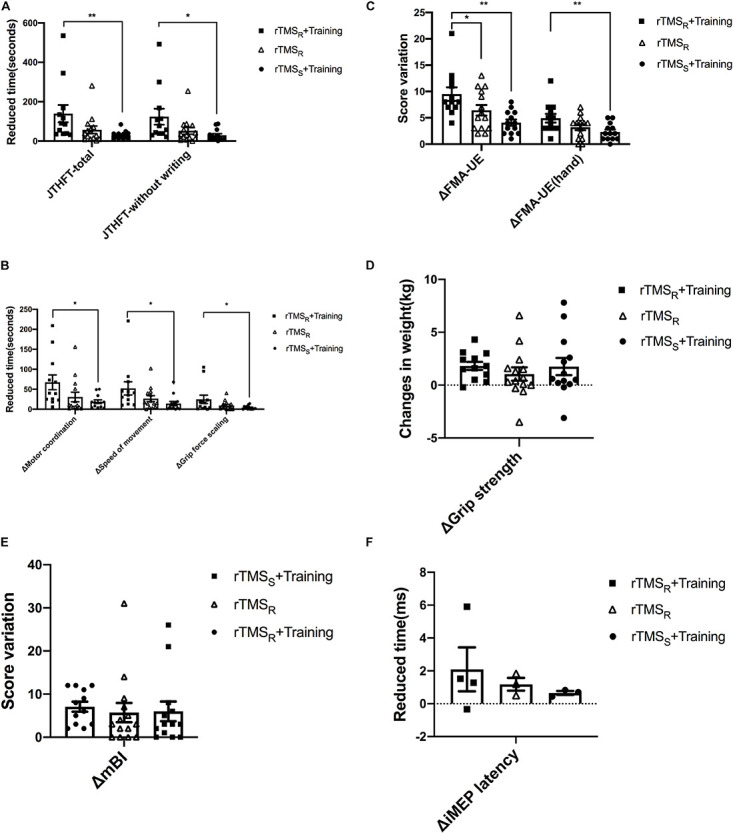
Behavioral and neurophysiological changes among the three groups (mean ± SEM). rTMS_R_+Training: rTMS and hand grip training (Group A); rTMS_R_: rTMS alone (Group B); rTMS_S_+Training: hand grip training alone (Group C). rTMS: repetitive transcranial magnetic stimulation. One-way ANOVA or nonparametric Kruskal–Wallis *H* test was used to compare the behavioral and neurophysiological changes among the three groups, and multiple comparisons with the whole pairwise comparation. *p < 0.05, **p < 0.01.

The change of JTHFT total score between baseline and post-intervention of the three groups (all *p* ≤ 0.01) were statistically significant (Table 2). Group A and group B also showed significant differences in JTHFT without writing, motor coordination, speed of movement, and grip force scaling (*p* < 0.05). Unfortunately, group C only showed significant difference in JTHFT without writing and motor coordination (*p* < 0.05), which, as mentioned above, indicated that both rTMS only and rTMS with hand grip training were effective in improving hand function in stroke patients. In addition, compared to group C, group A showed better enhancement in the JTHFT score (*p* < 0.05) (Table 3 and Figures 2A,B), suggesting that HF-rTMS combined with hand grip training was significantly greater than hand grip training only in hand function recovery among the stroke survivors.

**TABLE 2:** Behavioral outcome scores at baseline and post-intervention (mean ± SD).

	Group A		Group B		Group C	
	Pre	Post	Pre	Post	Pre	Post
**JTHFT (s)**						
JTHFT (total)	399 ± 256	259 ± 200**	323 ± 254	270 ± 235**	373 ± 238	344 ± 229**
JTHFT (without writing)	307 ± 235	183 ± 179**	256 ± 228	204 ± 216***	301 ± 215	271 ± 198***
Motor coordination	190 ± 103	123 ± 59**	146 ± 106	126 ± 89*	170 ± 100	155 ± 100*
Speed of movement	150 ± 111	103 ± 111**	132 ± 114	108 ± 117**	144 ± 109	133 ± 99
Grip force scaling	58 ± 47	33 ± 37**	45 ± 43	37 ± 40*	58 ± 45	56 ± 47
FMA-UE	47 ± 6	57 ± 5***	47 ± 8	53 ± 8***	47 ± 7	51 ± 7***
FMA-UE (hand)	16 ± 4	21 ± 3**	16 ± 5	19 ± 4**	15 ± 3	17 ± 4***
Grip strength (kg)	10 ± 5	12 ± 5***	11 ± 8	12 ± 28	11 ± 8	13 ± 9
mBI	89 ± 8	96 ± 26**	87 ± 213	93 ± 21**	78 ± 17	84 ± 15*

Group A, high-frequency rTMS during hand grip training; Group B, high-frequency rTMS alone; Group C, hand grip training alone; rTMS, repetitive transcranial magnetic stimulation. JTHFT, Jebsen–Taylor Hand Function Test; FMA-UE, Fugl-Meyer assessment of upper extremity; mBI, modified Barthel index; rTMS, repetitive transcranial magnetic stimulation. A paired *t* test or Wilcoxon rank-sum test was used to compare the values between baseline and post-intervention within each group. ***p ≤ 0.001 (compared to baseline). **p ≤ 0.01 (compared to baseline). *p < 0.05 (compared to baseline).

**TABLE 3:** Behavioral changes among three groups (mean ± SD).

	Group A	Group B	Group C	*p*	*p* (A vs B)	*p* (A vs C)	*p* (B vs C)
**ΔJTHFT(s)**							
ΔJTHFT (Total)	−140 ± 153**	−53 ± 75	−28 ± 23	0.006^b^	0.056^b^	0.006^b^	1.000^b^
ΔJTHFT (without writing)	−124 ± 140*	−52 ± 65	−29 ± 29	0.013^b^	0.113^b^	0.012^b^	1.000^b^
ΔMotor coordination	−55 ± 49*	−35 ± 71	−15 ± 20	0.029^b^	0.085^b^	0.041^b^	1.000^b^
ΔSpeed of movement	−47 ± 62*	−24 ± 29	−11 ± 22	0.035^b^	0.852^b^	0.031^b^	0.348^b^
ΔGrip force scaling	−25 ± 36*	−8 ± 12	−2 ± 6	0.035^b^	0.469^b^	0.029^b^	0.647^b^
ΔFMA-UE	10 ± 4**^#^	6 ± 4	4 ± 2	0.002^c^	0.035^c^	0.001^c^	0.095^c^
ΔFMA-UE (hand)	5 ± 3**	3 ± 2	2 ± 2	0.021^c^	0.062^c^	0.006^c^	0.302^c^
ΔGrip strength (kg)	2 ± 1	1 ± 2	2 ± 3	0.640^c^	0.406^c^	0.946^c^	0.436^c^
ΔmBI	7 ± 4	6 ± 8	6 ± 8	0.211^b^	>0.05^b^	>0.05^b^	>0.05^b^

Group A, high-frequency rTMS during hand grip training; Group B, high-frequency rTMS alone; Group C, hand grip training alone; rTMS, repetitive transcranial magnetic stimulation. A vs B, Group A compared with Group B; A vs C, Group A compared with Group C; B vs C, Group B compared with Group C; JTHFT, Jebsen–Taylor Hand Function Test; FMA-UE, Fugl-Meyer assessment of upper extremity; mBI, modified Barthel index; rTMS, repetitive transcranial magnetic stimulation. *p*^*b*^: nonparametric Kruskal–Wallis *H* test; *p*^*c*^: one-way ANOVA. **p < 0.01 (compared with Group C); *p < 0.05 (compared with Group C); ^#^*p* < 0.05 (compared with Group B).

#### Fugl-Meyer Assessment of Upper Extremity

Evaluation of FMA-UE and FMA-UE (hand) between baseline and post-intervention among the groups was significantly different (Table 2). The data showed that FMA-UE scores were greatly improved in all groups (*p* ≤ 0.01). Additionally, compared to group B (*p* < 0.05) and group C (*p* < 0.01), group A showed better potential in enhancing the score of FMA-UE and FMA-UE (hand) (Table 3 and Figure 2C), further suggesting that HF-rTMS with hand grip training was particularly effective in promoting hand and upper limb motor function in stroke patients with mild impairment.

#### Grip Strength

Significant improvement of grip strength was only found in group A after intervention (*p* ≤ 0.001), which indicated that HF-rTMS with hand grip training was capable of increasing power-grip force in stroke patients (Table 2). However, there were no significant differences found between groups in grip strength improvements (*p* > 0.05) (Table 3 and Figure 2D).

#### Modified Barthel Index

In the three groups, assessment of mBI revealed a significant increase at post- relative to pre-intervention (*p* < 0.05) in Table 2. However, no significant difference was detected between groups (*p* > 0.05) (Table 3 and Figure 2E).

### Neurophysiological Measures

#### iMEP Latency

The number of iMEP latency detected in these three groups is displayed in the Supplementary Material 1. Both group A and group C increased one patient in the detected number of iMEP. Notably, the iMEP latency shortened in all groups with no significant difference (*p* > 0.05) (Supplementary Material 2). In addition, there was similarly no statistical difference presented between groups (*p* > 0.05) (Figure 2F and Supplementary Table 3).

## Discussion

In this trial, we conducted a randomized and sham-controlled clinical research to detect the differential effects of HF-rTMS with hand grip training, HF-rTMS alone over the affected hemisphere, and hand grip training alone on the recovery of hand function in adult stroke patients suffering from mild impairment. From a clinical perspective, we found that all the three methods were beneficial to improve hand function and daily living ability in subacute adult stroke patients. Besides, we also detected that HF-rTMS with hand grip training method was superior to HF-rTMS alone and hand grip training alone. The results suggested that both central and peripheral stimulation were capable of enhancing hand functional recovery. Furthermore, our study provides evidence that the combination of central and peripheral stimulation could be the optimal approach for post-stroke rehabilitation and could increase the efficacy of rTMS.

Over the past decade, peripheral stimulation such as motor training was found favorable to promote injured cortical repair and remodeling *via* stimulating angiogenesis, neurogenesis, synaptogenesis, and dendritic plasticity (Zhang and Chopp, 2009). In recent years, numerous researches have been conducted to develop methods of improving the neurorehabilitation efficacy in stroke patients *via* non-invasive brain stimulation (Hummel and Cohen, 2006). rTMS, based on the principle of electromagnetic induction, induced the current generated by the coil placed over the surface of the skull to activate neurons in the cortical and subcortical regions, yielding neuronal depolarization (Kobayashi and Pascual-Leone, 2003). A single TMS stimulus could act on inhibitory or excitatory axons to depolarize them and deliver them backward. TMS induced changes in cell excitability and permeability (Ridding and Rothwell, 2007), which could influence cortical plasticity (Muller-Dahlhaus and Ziemann, 2015). Either rTMS or motor training could modulate neural plasticity and played a role in remodeling brain networks. In consequence, the hypothesis that the bond of rTMS and motor training might maximize their respective therapeutic effect had been proposed. Previous studies aiming to investigate the effect of combined rTMS and motor training were mainly time-locked, which referred to rTMS generally preceded or followed by motor training (Higgins et al., 2013; Kakuda et al., 2016). In recent several years, TMS was implemented during the procedure of motor training to further improve the efficacy of stroke motor function rehabilitation. These studies showed that coupling rTMS with motor training could have a synergic impact on motor recovery after stroke (Buetefisch et al., 2011; Massie et al., 2013a). The synergic effect could be explained by the rTMS-induced modulation of neural plasticity and the consolidation with motor training-induced (Buetefisch et al., 2011). Previous researches demonstrated that TMS plus motor training significantly improved the longevity of motor memory (Bütefisch et al., 2004) and hand function after stroke (Izumi et al., 2008). Another study revealed that functional rTMS (EMG-triggered rTMS) promoted greater excitatory changes and selectively modulated agonistic muscle activity (Massie et al., 2013a). Furthermore, consecutive multi-session functional rTMS could equally enhance cortical excitability and improve stabilities of motor skills (Massie et al., 2013b). However, the participants mainly included healthy subjects and chronic stroke patients, and the therapeutic frequency and intensity of rTMS were variable. Considering that the effect of rTMS on the cortical excitability is subject to intra-individual and inter-individual variability (Bestmann et al., 2010; Cohen et al., 2010), and that the optimal rTMS protocol is undetermined, developing more efficient clinical protocol that fits stroke patients with mild motor dysfunction is desirable and necessary. Our results provided a highly efficient approach to subacute stroke patients with mild dysfunction to promote hand function and useful experimental data for further larger-scale clinical trial.

Neuroplasticity presumably occurred in the connection between motor cortex neurons, which was naturally triggered during the period of muscle contraction, and simultaneously motivated over the motor cortex by rTMS (Edwardson et al., 2013). Considering our results from this standpoint, the rTMS plus hand grip training group was supposed to gain stronger excitability in the affected hemisphere. Unfortunately, the changes in the cortical excitability after HF-rTMS during hand grip training were not found. The following reasons may account for this result: the factors contributing to functional impairment included loss of white matter projection, diaschisis, and interhemispheric imbalance (Auriat et al., 2015). The detection method of recording iMEP latency was not able to find structural changes; other methods such as functional magnetic resonance imaging should be evaluated in future studies. According to the IHI model, which is based on interhemispheric imbalance, suppressing the unaffected hemisphere excitability or facilitating the affected hemisphere excitability could promote motor function recovery in stroke patients (Nowak et al., 2009). However, a novel model for neurorehabilitation named bimodal balance-recovery model suggested that the IHI model was oversimplified or even incorrect. The new model links interhemispheric balancing and functional recovery to the structural reserve spared by the lesion; it could be utilized to tailor treatment for individual patients (di Pino et al., 2014). Similarly, the small sample size could be unfavorable and has limited our study.

This trial was conducted safely with no seizures and other specific discomfort, and no incident happened. Thus, our results also provided additional evidence of the safety of implementing HF-rTMS during hand grip training and HF-rTMS alone in the clinical setting. Meanwhile, we concluded that HF-rTMS was an effective way to promote hand function in subacute adult stroke patients with mild impairment. In recent years, HF-rTMS has been reported to have a more effective impact when compared to low-frequency rTMS (LF-rTMS) in animal and human studies (Sasaki et al., 2013; Caglayan et al., 2019). An animal study detected that intervening 20-Hz HF-rTMS on acute and subacute ischemic injury models in mice induced excellent outcomes compared to the 1-Hz LF-rTMS. They simultaneously demonstrated that HF-rTMS decreased apoptosis and infarct volume; activated neurogenesis, neuronal survival, and neuronal plasticity; and increased regional cerebral blood flow. It has a strong support for the rationale of using HF-rTMS in post-stroke patients aiming at improving motor functional recovery (Caglayan et al., 2019). Besides, a clinical study targeted at stroke patients detected that 10-Hz HF-rTMS applied over the affected hemisphere induced significant improvement on motor functional recovery compared to the 1-Hz LF-rTMS (Sasaki et al., 2013). A meta-analysis revealed that facilitating affected M1 excitability could be directly beneficial compared to the suppressing unaffected M1 excitability in improving post-stroke recovery (McDonnell and Stinear, 2017). Future studies can focus on investigating the differential effect of LF-rTMS or HF-TMS plus hand grip training on hand functional recovery.

Though there was no significant difference detected between rTMS alone and hand grip training alone groups in FMA-UE score, the rTMS alone group showed enhancement in FMA-UE and the change had a minimal clinically important difference (Page et al., 2012). The short intervention period (10 sessions lasting 2 weeks) could explain the reason of no significant difference in grip strength among the groups (Stock et al., 2019). However, focusing on post-stroke patients with mild motor impairments, having no significant difference among these groups in mBI might be due to the sensitivity of the measurement.

The limitations of our study are as follows: (1) this study was a single-center trial and lacked sufficient subjects to administer subgroup analysis according to the lesion, stroke volume, the course of disease, and the severity of stroke; (2) we collected data to evaluate the short-term benefit from the proposed interventions, but we did not collect long-term data to assess the sustained benefits; and (3) the proportion of patients with positive MEP was lower than prior reported studies, and it limited our understanding of the mechanism of motor improvement by rTMS. Further larger-scale clinical trials are necessary to confirm our data and to promote this novel rehabilitation therapy.

## Conclusion

Our study demonstrated the short-term benefit of combined 10 sessions of 5-Hz rTMS over the affected hemisphere with concurrent hand grip training protocol. It provided important preliminary data to plan a large-sample, multi-center study to systematically evaluate the benefit of rTMS in a stroke population.

## Data Availability Statement

The original contributions presented in the study are included in the article/Supplementary Material, further inquiries can be directed to the corresponding author/s.

## Ethics Statement

The studies involving human participants were reviewed and approved by Institutional Ethics Review Board, Shanghai Ruijin Rehabilitation Hospital, Shanghai, China. The patients/participants provided their written informed consent to participate in this study.

## Author Contributions

JW and QX: conception and design of the study and data confirmation. YY: data collection, analysis of data, statistical analysis, and drafting the manuscript. HP: data segmentation and analysis of data. WP and YL: evaluating the patients and statistical analysis. XS and CN: acquisition of data. WF: analysis and interpretation of data. All authors contributed to the study and article and approved the submitted version. All authors contributed to the article and approved the submitted version.

## Conflict of Interest

The authors declare that the research was conducted in the absence of any commercial or financial relationships that could be construed as a potential conflict of interest.
