# Supplementary material for: Repetitive Transcranial Magnetic Stimulation on the Affected Hemisphere Enhances Hand Functional Recovery in Subacute Adult Stroke Patients: A Randomized Trial
Source: Front Aging Neurosci. 2021 May 19;13:636184. doi: 10.3389/fnagi.2021.636184 (PMC8171119; doi:10.3389/fnagi.2021.636184)
Supplement: Supplementary file 1 [file Table_1.docx]

Supplementary Material

|  | Group A | Group B | Group C | *p* |
| --- | --- | --- | --- | --- |
|  | n=12 | n=14 | n=13 |  |
| iMEP-Baseline | 4 | 3 | 3 | 0.761 |
| iMEP-post intervention | 5 | 3 | 4 | 0.537 |

**Supplementary Table 1 (S1).** The number of positive ipsilesional motor evoked potentials (iMEP).

Group A: high-frequency rTMS during hand grip training; Group B: high-frequency rTMS alone; Group C: hand grip training alone; rTMS: repetitive transcranial magnetic stimulation. iMEP: ipsilesional motor evoked potential latency.

Nonparametric Kruskal-Wallis H test was used to compare the number of positive iMEP among the three groups.

|  | Group A | Group B | Group C | *p* |
| --- | --- | --- | --- | --- |
|  | n=4 | n=3 | n=3 |  |
| iMEP-Baseline | 25.0$\pm$3.6 | 25.1$\pm$4.0 | 24.8$\pm$1.5 | 0.905 |
| iMEP-post intervention | 23.0$\pm$1.8 | 24.0$\pm$3.7 | 24.2$\pm$1.6 | 0.905 |

**Supplementary Table 2 (S2).** The latency of iMEP detected at baseline and post-intervention (ms).

Group A: high-frequency rTMS during hand grip training; Group B: high-frequency rTMS alone; Group C: hand grip training alone; rTMS: repetitive transcranial magnetic stimulation. iMEP: ipsilesional motor evoked potential latency.

Data are Mean±SD. Nonparametric Kruskal-Wallis H test was used to compare the iMEP latency among the three groups.

|  | **Group A** | **Group B** | **Group C** | ***p*** |
| --- | --- | --- | --- | --- |
|  | n=4 | n=3 | n=3 |  |
| ΔiMEP latency | $-$2.1$\pm$2.7 | $-$1.2$\pm0.7$ | $-$0.7$\pm$0.2 | 0.445 |

**Supplementary Table 3 (S3).** The latency change of iMEP among three groups (ms).

Group A: high-frequency rTMS during hand grip training; Group B: high-frequency rTMS alone; Group C: hand grip training alone; rTMS: repetitive transcranial magnetic stimulation. iMEP: ipsilesional motor evoked potential.

Data are Mean±SD. Nonparametric Kruskal-Wallis H test was used to compare neurophysiological changes among the three groups.
